# Correlation between neutrophil to lymphocyte ratio and C-reactive protein in diverse disease states in hospitalized patients

**DOI:** 10.12669/pjms.40.9.8933

**Published:** 2024-10

**Authors:** Ramsha Ghazal Arshad, Kaleem Ullah Toori

**Affiliations:** 1 Ramsha Ghazal Arshad, MBBS. Post Graduate Trainee Medicine, KRL Hospital, Islamabad, Pakistan; 2 Kaleem Ullah Toori. MBBS, FRCP. Head of Department Medicine, KRL Hospital, Islamabad, Pakistan

**Keywords:** C- reactive protein, Neutrophil to lymphocyte ratio, Diabetes, Malignancies, Stroke, Inflammation

## Abstract

**Objectives:**

This study aims to find if any significant correlation exists between C-reactive protein and Neutrophil to Lymphocyte Ratio as an indirect measure of inflammation.

**Methods:**

We selected 983 patients with any inflammatory condition who presented to a tertiary care hospital and were admitted in medical wards or Intensive Care Units (ICUs) of KRL Hospital Islamabad from December 2021 to December 2022. The study was a cross sectional study and convenience sampling was done. The patients were categorized into five groups depending upon their pathophysiology. Kolmogorov Smirnov test was used to assess the normality of the data, and Spearman’s coefficient was used to calculate the correlation between NLR and CRP.

**Results:**

A total of 983 patients were included. Mean CRP and NLR levels were 89.9±3.2 and 7.06±0.24, respectively. There was a significant positive correlation between CRP-NLR in the infectious, non-infectious non-inflammatory, and malignancy groups (0.420, 0.381, 0.642, *p* <0.01), and inflammatory group (0.322, *p* <0.05), and no correlation with chronic diseases.

**Conclusion:**

A significant correlation was shown to exist between CRP and NLR in patients with malignancies, non-infective non-inflammatory, inflammatory, and infective conditions and can therefore be used interchangeably to detect the presence of inflammation. Further exploration of these associations may contribute to a more nuanced understanding of the intricate relationships between different markers of inflammation and response to treatment.

## INTRODUCTION

C-reactive protein, a pentameric protein, is released by liver cells in response to both acute and chronic insults. It plays a documented role as both a pro-inflammatory and an anti-inflammatory substance. This multifaceted protein recognizes foreign entities as well as damaged innate cells, effectively eliminating them either directly or by activating the complement pathway. However, complications can arise when the same receptors are expressed on normal cells, such as autoantibodies, leading to prolonged immunological reactions as seen in conditions like SLE and other autoimmune disorders. It is a biomarker that has been extensively employed for evaluating the severity of ongoing inflammation. As an acute phase reactant, it is synthesized by the liver, and its regulation is influenced by proinflammatory cytokines like IL-6.^1^ This protein is released by macrophages and becomes detectable in the bloodstream within 6-10 hours following any tissue-damaging insult, subsiding over the course of 20 hours.

In contrast to the Erythrocyte sedimentation rate (ESR), CRP levels respond promptly to the extent and duration of injury. Consequently, they are considered a superior marker for gauging the degree of inflammation, making them a focal point of research in recent years. Nonetheless, despite these advancements, CRP levels still tend to lack specificity and remain unavailable in numerous healthcare centers. Alternative markers have been explored and compared with CRP to simplify clinical decision-making and management strategies

The Neutrophil to Lymphocyte Ratio (NLR) delineates the interplay between innate (neutrophils) and adaptive cellular immunity (lymphocytes) amidst pathological conditions.^2^ Investigating the correlation between these two biomarkers has been a subject of study across diverse disorders, yielding promising outcomes. There is potential for NLR to supersede CRP due to their independent significance. They hold value in diagnosing conditions, prognosticating the duration of hospitalization, and gauging treatment responses.^3^

Recent research has highlighted the significance of parameters based on blood cell counts, such as the Neutrophil to Lymphocyte Ratio (NLR), Platelet to Lymphocyte Ratio (PLR), and Lymphocyte to Monocyte Ratio (LMR), as remarkably sensitive biomarkers.^4^ These indices have demonstrated their utility in various areas, including disease prognosis. They have proven particularly valuable in prognosticating a range of conditions, including cancers,^5^ major cardiac events, and in predicting outcomes in cases of infectious^6^ or inflammatory disorders.^7^

Our study aimed to compare CRP and NLR levels, seeking to uncover any potential correlations between the two. While previous literature has primarily examined these parameters in isolation, typically within a single disease or a specific group of disorders when compared to a healthy population, our study took a more comprehensive approach: We included all patients admitted to the hospital, regardless of their condition, encompassing infectious, inflammatory, CNS pathologies, malignancies, and chronic diseases, and compared CRP and NLR levels across these diverse groups. As this was a cross-sectional study, data was obtained only at the time of admission in order to determine the baseline characteristics of the population and the original impact of the disease on the inflammatory process.

## METHODS

This was a single-center, cross-sectional, observational study that spanned over one year, from December 2021 to December 2022, conducted at Khan Research Laboratories (KRL) Hospital, Islamabad, Pakistan.

### Ethical Approval:

Institutional Review Board (IRB) approved the synopsis on 7^th^ December, 2021 (Ref ERC: KRL-HI-PUB-ERC/2020/35), before commencing and written informed consent was obtained, either from the patients or their legal guardians.

### Inclusion criteria:

Included all patients aged > 14 years, admitted with any inflammatory condition in medical wards or ICUs.

### Exclusion criteria:

Included patients needing surgical intervention or not meeting the inclusion criteria.

The patients were categorized into five distinct groups according to the diagnosis at the time of admission to the hospital: infective, inflammatory, non-infective non-inflammatory, chronic diseases, and malignancies. The inflammatory group included all the autoimmune conditions, non-infective non-inflammatory included new onset Transient ischemic attacks, cerebrovascular accidents and anemia, and chronic diseases included diabetes mellitus, hypertension, ischemic heart disease and decompensated liver diseases.

Upon admission, demographic characteristics such as age, gender and existing medical conditions were documented. Additionally, comprehensive baseline assessments were conducted, including complete blood counts, liver and renal function tests, electrolyte levels, in addition to CRP measurements. CRP levels were taken as raised >5mg/L and neutrophil to lymphocyte ratio was calculated by dividing neutrophils to lymphocytes. A *p* value of <0.05 with a confidence interval of 95% was taken to be significant.

### Statistical Analysis:

A total of 983 patients were enrolled in the study using convenience sampling over the course of one year. Frequencies were computed for qualitative variables such as gender and, the Kolmogorov-Smirnov test was employed to assess the normal distribution of data. This analysis yielded values of 0.184 for CRP and 0.191 for NLR, both with a *p-*value of <0.01 which effectively contradicted the null hypothesis that the data is distributed normally. Spearman’s correlation co-efficient *rho* was used to assess the correlation between CRP and NLR within all the subgroups.

## RESULTS

Of the total 983 patients, 582 (59%) were males and 401 (41%) were females. The mean age of males was 55±8.2 and that of females was 57.8±16.2. There were 667 cases (67.9%) that were classified as infective, 51 cases (5.2%) categorized as inflammatory, the non-infective non-inflammatory group contained 187 cases (19%), 37 cases (3.8%) in the malignancy group and 41 cases (4.2%) were related to chronic diseases.

**Fig.1-5:**
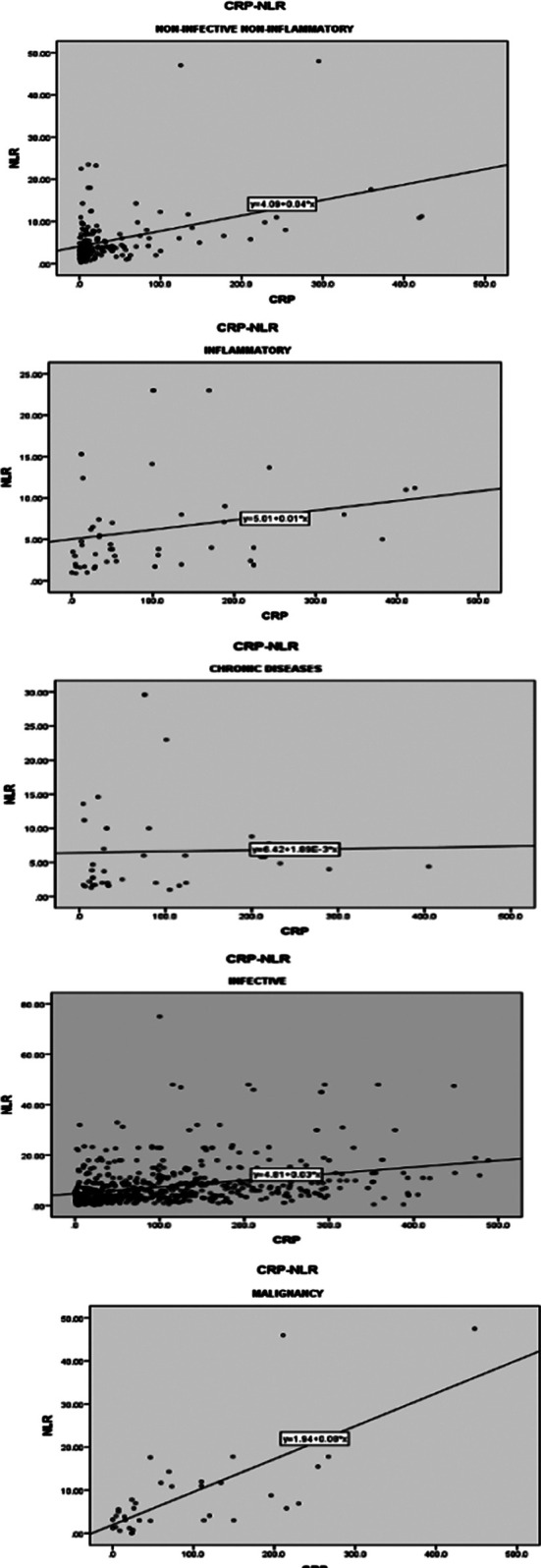
Correlation between CRP and NLR in distinct groups Fig.1: Correlation between CRP and NLR. in non-infective, non-inflammatory group. Fig.2: Correlation between CRP & NLR in inflammatory group. Fig.3: Correlation between CRP and NLR in Infectious group. Fig.4: Correlation between CRP and NLR in Malignancies. Fig.5: Correlation between CRP and NLR in chronic diseases.

The mean value of CRP was 89.9 + 3.2 (83.7 to 96.2) and of NLR was 7.06 + 0.24 (6.8 to 7.3) overall. The mean values of CRP, NLR, and other demographic characteristics are listed in Table-I. The analysis revealed a non-significant correlation of CRP and NLR with chronic diseases (rho 0.193, *p=*0.233), and a significant correlation with malignancies (rho 0.642, *p*<0.01), non-infective non-inflammatory (rho 0.381, *p*<0.01), inflammatory (rho 0.322, *p*=0.021) and infective (rho 0.420, *p*<0.01) conditions. (Table-II, Fig.1-5)

**Table-I:** Baseline characteristics of the study population.

	Infective	Inflammatory	Non-infective non- inflammatory	Malignancies	Chronic diseases
Gender (Males/Females)	400/267	33/18	101/86	21/16	26/15
Age in years (SD)	56.6(17.1)	52.2(19.2)	54.6(18.2)	62.4(18.7)	54.2(18.4)
CRP (SD)	105(101.4)	100.1(111)	34.7(66.9)	89.4(100.2)	83.4(93.4)
NLR (SD)	7.6(8.0)	6.2(5.8)	5.3(5.9)	8.8(10.6)	6.5(6.9)

**Table-II:** Correlation between CRP-NLR Spearman’s coefficient (rho).

	Infective	Inflammatory	Non-infective non-inflammatory	Malignancies	Chronic diseases
CRP-NLR	0.420*	0.322**	0.381*	0.642*	0.193

* Significant at <0.01 (2-tailed),

** Significant at <0.05 (2-tailed).

## DISCUSSION

In this study, correlation between CRP and NLR was identified in diverse groups admitted to the hospital. Amongst these, the infective, inflammatory, those suffering from neurological diseases and malignancies showed a significant correlation, indicating a possible interplay between different measures of inflammation and a potential for them to be used interchangeably in limited resources conditions.

In our study a positive correlation was seen between CRP and NLR within the infectious group (rho 0.420, *p<0.01)*. This group, comprising 667 patients, represented the largest number of individuals in our study. Additionally, it had the highest recorded CRP value (105+101.4). This underscores the significance of this group within the study and highlights the wide spectrum of cases it encompassed. These findings were substantiated by prior literature in which a retrospective study showed that NLR values were higher in pneumonic COVID-19 patients compared with patients with non-pneumonic COVID-19 infection. NLR was positively correlated with CRP (r=0.385 *p*<0.001), and procalcitonin (r=0.238, *p*<0.001).^8^ These findings were substantiated by another recent study showing a positive correlation between IL-8, NLR, CRP and mortality in COVID-19 patients with DM comorbidity.^9^ Ragolo et al, showed that NLR levels were significantly higher in Covid-19 patients who were admitted in ICU care, however, that was independent of any demographic characteristics or comorbid conditions.^10^ Similarly, another study predicted an infection diagnosis with a sensitivity of 69% and a specificity of 84% with an OR of 25.59 (95% CI: 9.73–67.31).^11^

Li et al., showed that NLR is an important independent risk factor for maintenance hemodialysis in patients with pulmonary infection,^12^ while Carvallho et al., studied the association of CRP with NLR and PLR, which showed the values of sensitivity and specificity for the prediction of infection equal to 67% and 67%, 65% and 58%, and 71% and 53%, respectively. 13

In the inflammatory group, our study included patients admitted with various autoimmune conditions and their complications, excluding infections. These patients represented a diverse range of age groups and notably included individuals with active or uncontrolled disease, irrespective of treatment regimens and yielded results (rho 0.322, *p*<0.05), which were in accordance with the studies conducted internationally.^7^,^14^,^15^

A meta-analysis showed that, cirrhotic patients with ascites who developed SBP had elevated levels of NLR compared to those who did not,^16^ while another study on CRP and NLR levels in patients with Hepatocellular carcinoma (HCC) to investigate their potential role in guiding treatment response showed that patients with elevated CRP and NLR had a significantly shorter overall survival than those with low CRP and low NLR (all *p* < 0.001) and the combined use of CRP and NLR provided incremental prognostic information.^5^ Another study found out that CRP/albumin, NLR, PLR, and RDW values were statistically significantly higher in patients with severe acute pancreatitis compared to those with mild acute pancreatitis according to the BISAP score (p<0.001).^17^

In the malignancy group, our study yielded a notably positive outcome, consistent with prior research,^18^-^20^ despite this subgroup being relatively small, consisting of only 37 patients. However, these patients tended to be older (mean age 62.4 + 18.7), and they exhibited the highest average NLR value among all the subgroups. The correlation coefficient (rho) value of 0.642 observed within this subgroup was also statistically significant at *p*<0.01, marking the highest correlation value among all the subgroups studied.

In our study, we included a total of 187 patients in the non-infective, non-inflammatory group. A positive correlation (rho 0.381, *p*<0.01) was observed within this subgroup as well. A study showed that in GBS patients, the mean CRP and NLR levels at admission/discharge and third-month control were significantly higher.^21^ Another study showed that higher NLR levels were significantly associated with higher NIHSS scores (*p* = 0.011) and unfavorable outcomes in ischemic stroke patients.^22^

However, it is important to note that this group encompassed a wide range of cases, including those with hematological, neurological and other conditions not specified to the other groups. This diversity could potentially have limited our ability to measure the correlation specifically within each of these individual disease entities. This group also exhibited the lowest values of both CRP and NLR (34.7 + 66.9, 5.3 + 5.9 respectively) in comparison to other groups in our study. This further emphasizes the heterogeneity within this group, spanning various medical conditions with distinct inflammation profiles.

In the chronic disease’s subgroup, our study revealed no significant correlation amongst chronic diseases like T2DM, Hypertension or chronic liver diseases which was in contrast to the published literature in different chronic ailments.^22^,^23^ This might have been due to lesser number of patients included in the study (41 patients) in this group.

### Limitations:

Despite these encouraging results, it is important to acknowledge the limitations of our study. This was a single center cross-sectional study and we only measured CRP and NLR levels at admission, without employing follow-up tests to assess treatment responses. Additionally, our sampling process involved recruiting all eligible patients who were readily available which could introduce potential selection bias.

## CONCLUSION

This study highlights a notable association between CRP and NLR across various subgroups of the population, suggesting that they can be used interchangeably as straightforward measures of the degree of inflammation or injury. This could prove valuable especially in settings with limited resources, as it offers a practical approach to assessing inflammatory responses.

### Author`s Contribution:

**RGA:** Data collection, analysis and write up.

**KUT:** Revision of the article and approval before final submission.
